# Mechanisms of *E. coli* chemotaxis signaling pathways visualized using cryoET and computational approaches

**DOI:** 10.1042/BST20220191

**Published:** 2022-11-24

**Authors:** Kyprianos Hadjidemetriou, Satinder Kaur, C. Keith Cassidy, Peijun Zhang

**Affiliations:** 1 Diamond Light Source, Harwell Science and Innovation Campus, Didcot OX11 0DE, U.K.; 2 Division of Structural Biology, Wellcome Trust Centre for Human Genetics, University of Oxford, Oxford OX3 7BN, U.K.; 3 Chinese Academy of Medical Sciences Oxford Institute, University of Oxford, Oxford OX3 7BN, U.K.

**Keywords:** chemoreceptor, chemosensory array, chemotaxis, core signaling complex, cryo-electron tomography, cryoEM

## Abstract

Chemotaxis signaling pathways enable bacteria to sense and respond to their chemical environment and, in some species, are critical for lifestyle processes such as biofilm formation and pathogenesis. The signal transduction underlying chemotaxis behavior is mediated by large, highly ordered protein complexes known as chemosensory arrays. For nearly two decades, cryo-electron tomography (cryoET) has been used to image chemosensory arrays, providing an increasingly detailed understanding of their structure and function. In this mini-review, we provide an overview of the use of cryoET to study chemosensory arrays, including imaging strategies, key results, and outstanding questions. We further discuss the application of molecular modeling and simulation techniques to complement structure determination efforts and provide insight into signaling mechanisms. We close the review with a brief outlook, highlighting promising future directions for the field.

## Introduction

Bacterial chemotaxis pathways couple the sensing of environmental chemical gradients to changes in cellular motility and are the best-understood biological signaling systems owing to over half a century of investigation [1–3]. Moreover, the roles played by chemotaxis in other important processes governing bacterial lifestyle, including biofilm formation, host–microbe interactions, and pathogenesis, are becoming increasingly appreciated [4,5]. As such, chemotaxis pathways are central to our understanding of basic microbial signaling and behavior and a detailed understanding of signal transduction mechanisms within the comprising proteins promises to provide novel strategies for affecting bacterial behavior in medicinal and technological applications.

**Figure 1.**
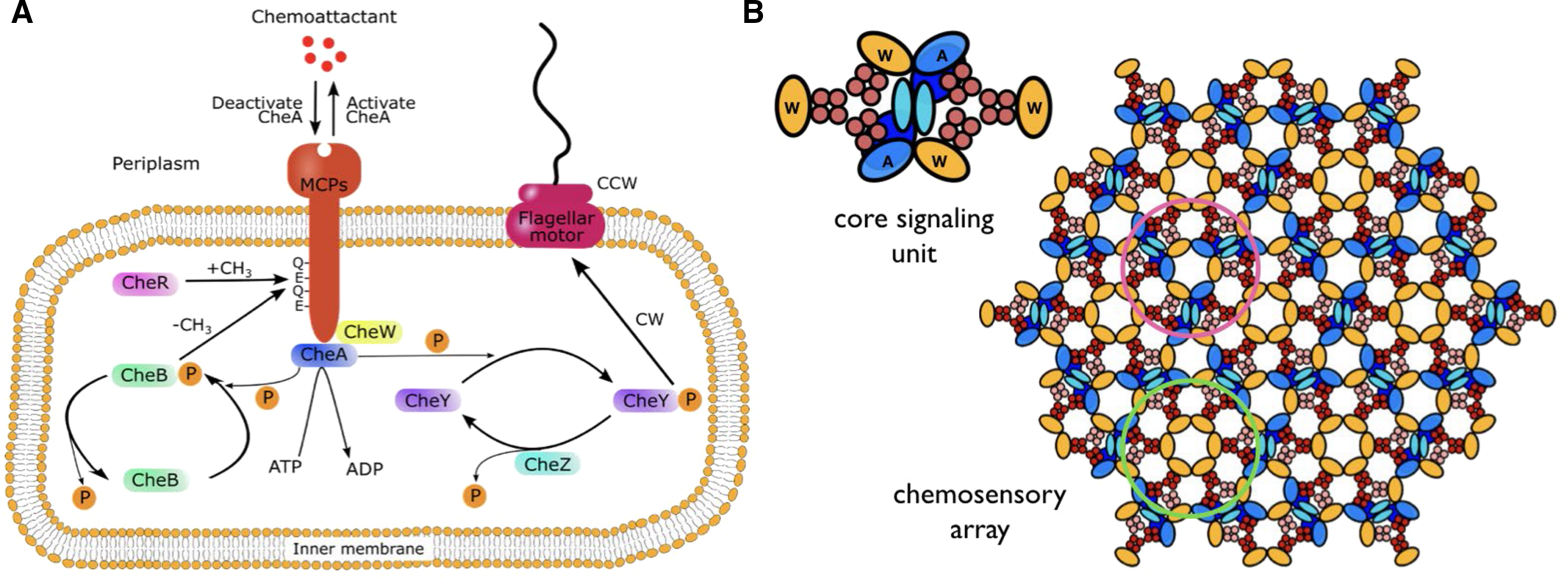
*E. coli* chemotaxis signaling. (**A**) Schematic of the *E. coli* chemotaxis signaling pathway, highlighting component interactions connecting the periplasmic chemical environment to the direction of flagellar motor rotation as described in the main text. (**B**) Schematic of the *E. coli* chemosensory array, shown as the level of the array baseplate, illustrating the hexagonal arrangement of receptor trimers-of-dimers (TOD) interconnected by rings of CheA and CheW (pink circle) and CheW only (green circle). A single core signaling unit, along with two flanking CheW molecules, is isolated to the left. Chemoreceptors are shown in red and pink, CheA in shades of blue, and CheW in gold. Panel (**B**) was adapted from [52] with permission.

The study of chemotaxis pathways has historically largely been conducted in the model organism *Escherichia coli*, owing to its relative tractability in terms of the number of signaling components. An overview of the *E. coli* chemosensory pathway is provided in Figure 1A. Briefly, the signaling state of a chemoreceptor (red) is altered through the binding of chemoeffectors, which mediates the autophosphorylation activity of an associated CheA kinase (blue). CheW (yellow) is required for the functional coupling between receptors and CheA. Upon activation, CheA transfers a phosphoryl group from bound ATP to the response regulator CheY (dark purple), which in turn becomes activated (CheY-P) and can bind to the flagellar motor (magenta) to alter its direction of rotation. The direction of motor rotation determines whether the cell ‘runs’ (swims straight) or ‘tumbles’ (changes to a new, random direction). Thus, by controlling the relative amount of CheY-P through the receptor-mediated regulation of CheA activity, environmental chemical information is coupled to the swimming pattern of the cell. The CheA signal is eventually terminated by the removal of phosphate groups from CheY-P by CheZ (teal). Additionally, an adaptation system is provided by CheR (pink) and CheB (green), which add and remove methyl groups, respectively, at specific sites on the receptor to tune its response to ligand binding. An increase in receptor methylation increases CheA autophosphorylation and vice versa. Although CheR is constitutively active, the rate at which CheB removes methyl groups is increased by phosphorylated via CheA, giving rise to a negative feedback loop that resets the pathway to baseline activity.

The structures and signaling mechanisms of chemoreceptors and CheA have been studied intensely [3,6]. Both are modular, multi-domain proteins, largely utilizing subtle allosteric changes to transduce sensory signals. Canonical chemoreceptors are long (∼30 nm), mostly helical homodimeric proteins. They sense chemicals using a periplasmic ligand-binding domain and initiate signals passing through a transmembrane four-helix bundle and on to a cytoplasmic HAMP domain. After the HAMP domain, the signal is perceived by a cytoplasmic coiled-coil domain containing three distinct modules: the methylation-helix bundle where adaptational modification occurs, a flexible bundle containing the glycine hinge, and a signaling tip containing the sites for CheA and CheW binding as well as receptor trimerization. CheA is a homodimeric histidine autokinase with each protomer containing five distinct domains (P1–P5) connected by flexible linkers of varying lengths. The dimerization (P3), ATP-binding (P4), and regulatory (P5) domains form a catalytic core, which integrates into the array through interactions with receptors and CheW. The P1 and P2 domains are connected to the P3P4P5 core through longer disordered linkers. The former domain contains the substrate histidine, which accepts a phosphoryl group from an ATP molecule bound to P4, and the latter binds the response regulator to facilitate the further transfer of the phosphoryl group.

Central to the chemotaxis response is the clustering of thousands of copies of chemoreceptors, CheA, and CheW into large, transmembrane signaling complexes known as chemosensory arrays (Figure 1B) [3,7]. The functional unit of the chemosensory array, known as the core-signaling unit (CSU), contains six receptor dimers, organized two receptor trimers-of-dimers (TOD), a single CheA dimer, and two or four CheW monomers (Figure 1B) [8]. The CSU is the minimal complex required to place CheA autophosphorylation under receptor control, allowing it to be altered by ligand binding and adaptational modification [8]. Neighboring CSUs are structurally and functionally coupled through an interlocked ‘baseplate’ formed by hexameric rings containing the CheA regulatory domain and CheW, while two additional flanking CheW molecules can associate with the CSU to produce hexameric rings containing only CheW [9,10]. Importantly, the highly ordered and interconnected nature of chemosensory arrays enables long-range cooperative responses, which give rise to impressive signal integration, amplification, and adaptation features that are distinctive of chemotaxis pathways [11–14]. To understand chemotaxis signaling, therefore, a detailed description of signaling within and between CSUs is required.

Despite impressive results using techniques such as X-ray crystallography and NMR spectroscopy, high-resolution information on the CSU has mostly been limited to individual domains and sub-complexes due to its large size and dynamic nature. Cryo-electron tomography (cryoET) provides a powerful means to study large and heterogeneous biomolecular complexes such as chemosensory arrays that are intractable using other structure determination techniques [15–17]. In this mini-review, we will provide a broad overview of the use of cryoET to study bacterial chemosensory array structure and function, including imaging strategies, key results, and outstanding questions. We will further highlight the application of molecular modeling and simulation techniques to complement structure determination efforts and provide mechanistic insight, followed by a brief discussion on the outlook of the field. Finally, although the focus of the present review is on chemosensory arrays, we wish to note here the recent use of cryoET to study the ‘output end’ of chemotaxis pathways, namely the bacterial flagellar motor, which has lately provided insight into the mechanisms of CheY signaling as well as motor assembly and control [18–22].

## CryoET analysis of bacterial chemosensory arrays: an overview

**Figure 2.**
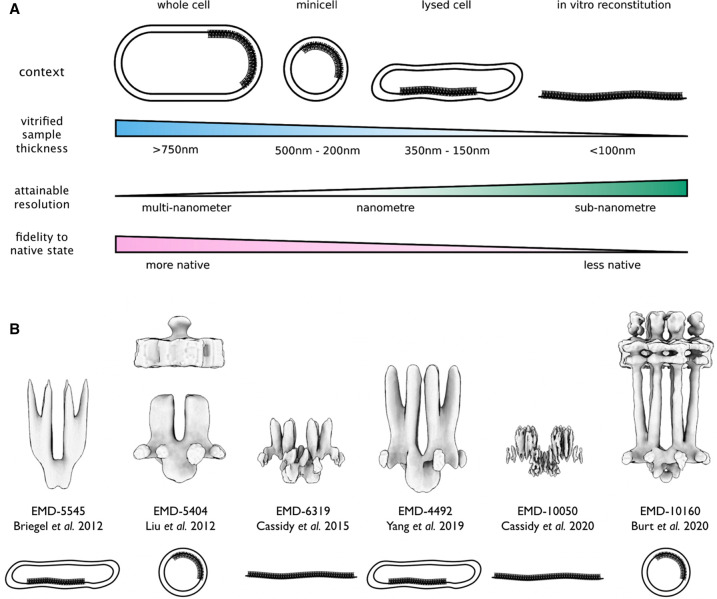
Overview of cryoET applications to chemosensory arrays. (**A**) Schematics of the primary imaging contexts used to visualize chemosensory arrays. A range of vitrified sample thicknesses and attainable resolutions are given for each context. In general, as the thickness of a vitrified sample decreases, the attainable resolution increases, but often at the expense of fidelity to the native environment. (**B**) Renderings of representative core signaling unit (CSU) structures published in key studies and shown in chronological order from left to right. The EMDB accession code and associated imaging context are also indicated. Figure panels were kindly contributed by Dr. Alister Burt.

Over the years, chemosensory arrays have been imaged using cryoET in multiple biological contexts, which exploit the trade-off between sample thickness and attainable resolution, while attempting to remain as true to their native environment as possible (Figure 2A) [23]. Whole cells were first used to obtain images of extended zipper-like chemoreceptor structures, resulting from receptor overexpression [24,25]. Shortly thereafter, the first images of intact chemosensory arrays in *E. coli* [26] and *C. crescentus* [27] were obtained, establishing their overall makeup and hexagonal architecture. Briegel and co-workers subsequently demonstrated that this hexagonal array architecture was conserved across a wide range of bacteria and archaea [28,29], suggesting the broad conservation of a basic chemotaxis signaling mechanism. Given the thickness of most cells, however, the resolutions obtainable when imaging whole cells are typically limited to 30–40 Å, preventing the separation of individual proteins within the resulting density maps.

To produce increasingly detailed images of chemosensory arrays, several strategies have been developed to reduce the thickness of samples before imaging (Figure 2A). The most common approach is the use of lysed cells, in which treatment with penicillin or lysozyme [9,30] or the introduction of an inducible phage E-gene [31] is used to leak the cell's cytoplasmic content. Additionally, manipulation of the cellular division machinery can be used to produce array-containing minicells [10,32–35], for instance, through mutations to the Min system which cause budding near the cell poles. The lysed cell and minicell approaches have the benefit of improving resolutions to 15–30 Å, or even higher [36], while maintaining the complete chemosensory complex in a near-native membrane environment. For high-resolution structure analysis, an *in vitro* reconstituted system has also been developed, which involves the use of purified recombinant *E. coli* Tar cytoplasmic fragments (TarCF), CheA, and CheW to form the array lattice on a 2D lipid monolayer [37,38]. This system offers several additional advantages, including a well-defined composition and large patches of well-ordered arrays, that permit higher resolutions (8–15 Å). However, receptor truncation limits the obtainable functional information to the baseplate region. Thus, each imaging context has its inherent strengths and weaknesses and a complete structural picture of the chemosensory array is likely to require the consolidation of insights from each. Although beyond the scope of this review, it's important to note that increases in resolution have also been greatly helped by advances in hardware and image processing, particularly sub-tomogram averaging (STA) techniques for which repetitive structures like chemosensory arrays are ideally suited [39].

Figure 2B highlights several structural milestones along with their associated imaging contexts. Early cryoET studies in *E. coli* employed lysed cells (EMD-5545) [9] and minicells (EMD-5404) [10] to establish a molecular picture of the array baseplate, namely a hexagonal lattice of receptor TODs interconnected by hexameric rings involving CheA and CheW (Figure 1B). Building on this work, subsequent studies in lysed cells imaged chemosensory arrays carrying receptor mutations designed to induce distinct signaling states, enabling the observation of concerted structural changes in CheA [40] and the cytoplasmic region of the receptor TOD (EMD-4492) [30], albeit at resolutions of 20 Å or lower. Use of the *in vitro* monolayer system considerably improved the resolution of the baseplate region to ∼12 Å, providing unambiguous visualization of the CheA dimerization (P3) and catalytic (P4) domains as well as CheW (EMD-6319) [37]. Despite these advances, the membrane-proximal regions of the complex remained unresolved until a recent study from Burt and co-workers reported the first full-length CSU structure from *E. coli* minicells (EMD-10160) [33]. At resolutions of mostly between 15–30 Å, the structure exhibits continuous receptor densities, including for the HAMP, transmembrane, and ligand-binding domains, although with a weaker density. At the same time, improvements in image processing have allowed the CSU baseplate to be imaged to sub-nanometer resolution using the *in vitro* monolayer system (EMD-10050) [38], revealing individual helices within the receptor signaling tips and beta-barrel structures within the CheA regulatory domain (P5) and CheW, thereby, precisely setting their interaction interfaces. Taken together, these results form a complete basic picture of CSU structure in *E. coli*, providing a ground state for testing mechanistic hypotheses. As described below, such data can be further combined with computational techniques to yield high-fidelity molecular models of the CSU and extended array.

## Organizational variability in chemosensory arrays

As previously noted, much of our structural understanding of chemosensory arrays comes from *E. coli* due both to its relative simplicity, in terms of the number of pathway components, and our ability to readily manipulate its physiology genetically. Nevertheless, most motile bacteria possess multiple, more complex, chemosensory pathways operating simultaneously and controlling a wide range of responses [41–44], implying a richness of chemosensory machinery. Indeed, despite the apparent ubiquity across species of the hexagonal architecture of transmembrane chemosensory arrays at the receptor level [28,29], recent cryoET studies are revealing a diversity of baseplate compositions and organizations [45–47] as well as the existence of cytoplasmic chemosensory arrays that are essential for normal chemotaxis [48,49]. These topics have recently been covered comprehensively in an excellent review by Yang and Briegel [50].

Here we will detail one particularly intriguing example of array variability: the recent observation of an alternative array architecture in *Treponema denticola* [51] and *E. coli* minicells [52], which differs markedly from the canonical one observed in native *E. coli* cells. Indeed, although the alternative and canonical architectures appear the same at the level of the receptors, they differ considerably in baseplate organization: the canonical architecture contains two types of baseplate hexameric rings, namely (A.P5/W)_3_ and (W)_6_, with CheA organized on into a kagome lattice, whereas the alternative architecture has only a single type of baseplate ring, namely (A.P5/W/W)_2_, and CheA is organized into linear stripes [51,52]. Thus, although the CSU itself remains unaltered, the flanking CheW molecules take on an essential role in the formation of the alternative architecture, whereas in the canonical case they reinforce an existing lattice [52,53]. As the relative abundance of (W)_6_ rings in native *E. coli* arrays affects response cooperativity [53], the two architectures may therefore possess different inherent cooperativities due to their varying CSU connectivity. Comparative studies, therefore, might provide a potential means to investigate certain emergent signaling features of the chemotaxis pathway [11,12].

What factors might give rise to this alternative architecture? In the case of the *T. denticola* arrays, in which the characteristic stripes of CheA were observed to align with the cell axis, it was proposed that the observed architecture might be necessary to accommodate high membrane curvature [51]. Observations of the same architecture in *E. coli* minicells, which are considerably less curved than *T. denticola* cells, as well as in flat *in vitro* monolayer arrays suggest additional factors may also be at play [52]. For instance, the differential role of the flanking CheW molecules may suggest the relative expression levels of the different array components could favor one organization over the other [52,54]. Nevertheless, it seems clear that the membrane plays a considerable role in array assembly, suggesting that these observations may be related to another long-standing question in the field, namely the mechanism of polar localization. Although poorly understood, polar localization in various systems has been linked to membrane curvature and cardiolipin concentration [55,56], complex interactions with the ParA/MinD family [57] and TOL/PAL complexes [58], and the inherent geometry of receptor TODs [59]. CryoET provides a unique and promising tool to make headway on these important issues.

## Integrative modeling and molecular simulation of chemosensory arrays

Despite the remarkable insights into chemosensory array structure gained by cryoET, resolutions obtained have so far been limited to ∼8 Å, but are generally much lower for membrane-embedded arrays (15–30 Å), preventing the *de novo* assignment of atomic structure to the density map. The use of integrative modeling and molecular dynamics (MD) simulation techniques has therefore been necessary to extend the chemical interpretability of obtained cryoET structures, enabling domain- and residue-level insights not possible otherwise. Early studies relied on rigid docking to position high-resolution structures, obtained via X-ray crystallography, NMR, or homology modeling, within a density map, providing an overall picture of array organization and rough interaction interfaces between array components [9,10]. As resolutions improved, flexible refinement techniques such as Molecular Dynamics Flexible Fitting (MDFF) [60,61] provided a more robust interpretation of cryoET densities. Such techniques use MD simulation to optimize the conformations of high-resolution structures using a potential generated explicitly from a density map. MDFF has been applied to flexibly refine receptor TOD models into *E. coli* maps corresponding to distinct signaling states, highlighting concerted changes in the mobility of the P1 and P2 domains of CheA [40] and suggesting that TODs may undergo transitions between an expanded and compact conformation during signaling, enabled by receptor bending at the HAMP domain and/or glycine hinge [25,30,62].

**Figure 3.**
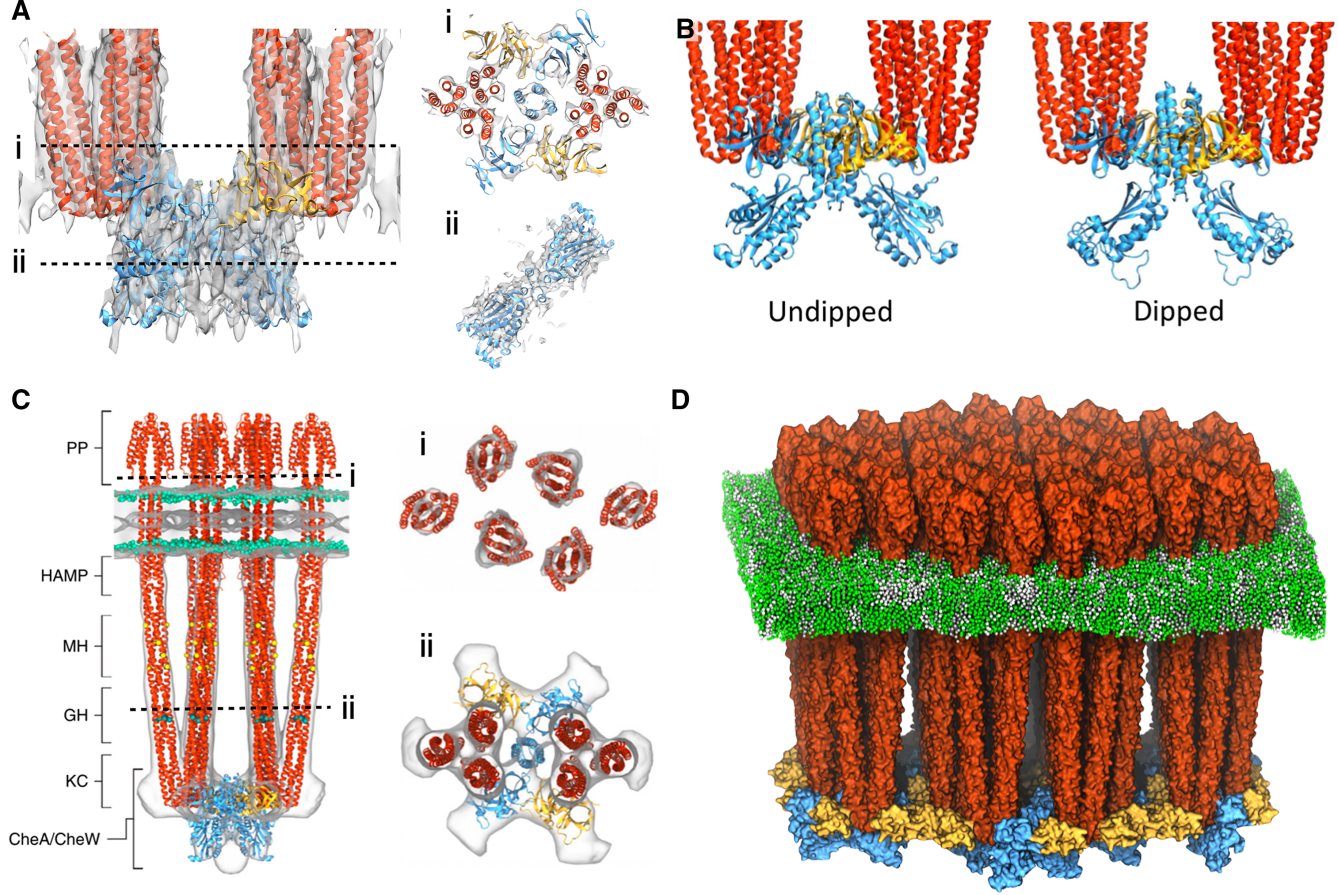
Recent models of chemosensory array structure. (**A**) Overlay of 8.4 Å *in vitro* monolayer map (EMD-10050) and resulting all-atom model of the *E. coli* core-singling unit (CSU) baseplate (PDB 6S1K), shown from the side (left) and the top as slices through the baseplate (right, top) and CheA.P4 domains (right, bottom). (**B**) Undipped (left) and dipped (right) conformations obtained from computational analysis of CheA.P4 conformational landscape. (**C**) Overlay of *E. coli* minicell map (EMD-10160) and resulting all-atom model, shown from the side (left) and the top as slices through the periplasmic domains (right, top) and baseplate (right, bottom). (**D**) All-atom model of the transmembrane chemosensory array, assembled from the CSU model in (**C**). In all panels, chemoreceptors are colored in red, CheA in blue, and CheW in gold. Panels (**A**) and (**B**) were adapted from [38] with permission; panel (**C**) was adapted from [33] with permission.

The application of computational modeling has been especially important for the construction of all-atom models based on *in vitro* monolayer cryoET data [37,38]. Here, overall resolutions allow for reliable placement of individual protein domains, however, conformational heterogeneity within the sample results in structural ambiguities that require systematic assessment. Initial efforts used existing X-ray crystallography structures from *T. maritima* along with MDFF to construct an all-atom model of the cytoplasmic portion of a chemosensory array (PDB 3JA6) [37]. Subsequent large-scale MD simulations identified a novel conformational change in CheA, namely the dipping of the catalytic P4 domain, as well as critical stabilizing residues. Building on these results, an all-atom model of the *E. coli* CSU baseplate (PDB 6S1K) was constructed in a follow-up study using higher-resolution *in vitro* monolayer cryoET data and homology modeling (Figure 3A) [38]. Again, all-atom MD simulations were used to interrogate the conformation landscape of CheA, identifying two classes of CheA conformation, namely an undipped and dipped state (Figure 3B) analogous to those identified previously [37], and highlighted structural changes in the critical flexible linker connecting the P3 and P4 domains [38,63]. These computational predictions have since been corroborated by multiple studies using different techniques [64–66]. Additionally, modeling has been used to gain insights into CheA function, particularly the potential placement of the P1 and P2 domains, through the construction of models of CSU-like *T. maritima* receptor fold-on complexes using molecular envelopes from Small Angle X-ray Scattering (SAXS) informed by disulfide cross-linking and ESR spectroscopy data [65].

Most recently, an all-atom model of the full-length *E. coli* transmembrane CSU was presented (Figure 3C) [33]. Consisting of homology models for CheA and CheW as well as a full-length model of *E. coli* Tsr constructed using existing X-ray crystal structures and disulfide cross-linking data, the model was conformationally refined to cryoET data from *E. coli* minicells using MDFF. Given that the CSU is the minimal complex required for basic receptor-mediated kinase control [8], such a model provides a potential workhorse for the *in silico* investigation of signaling mechanisms. Previous all-atom MD simulations of isolated chemoreceptor fragments have provided insight into signal transduction within the transmembrane domain [67,68] and cytoplasmic domains [69,70], while coarse-grained simulations have been used to study overall receptor dynamics [71,72] and the effects of adaptational modification [73]. Given the CSU's size (millions of atoms) and complexity as well as the subtlety of the allosteric changes underlying signaling, large-scale all-atom simulations on the microsecond timescale are required to investigate signaling in sufficient detail. While such simulations are not yet routine, recent advances in computing power as well as optimized MD codes render them no longer prohibitive [74–76]. Additionally, coarse-grained simulations, using for example the Martini [77] or shape-based [78] approaches, provide a means to study even larger, multi-CSU models (Figure 3D), particularly membrane-related phenomena [79,80]. Finally, with the recent development of AlphaFold2 [81,82], high-quality models of full-length sensory proteins (e.g. chemoreceptors and CheA) can be constructed given only a sequence. Historically, high-resolution experimental structural information has been sparse, outside of a few organisms. AlphaFold2 thus provides an efficient means to expand the structural repertoire amenable to molecular simulations. The combination of cryoET, AlphaFold2, and molecular simulations, therefore, represents a powerful multi-scale tool for interpreting a wide range of existing data and designing further experiments to understand signaling mechanisms in diverse species.

## Outlook for cryoET analysis of chemosensory arrays

**Figure 4.**
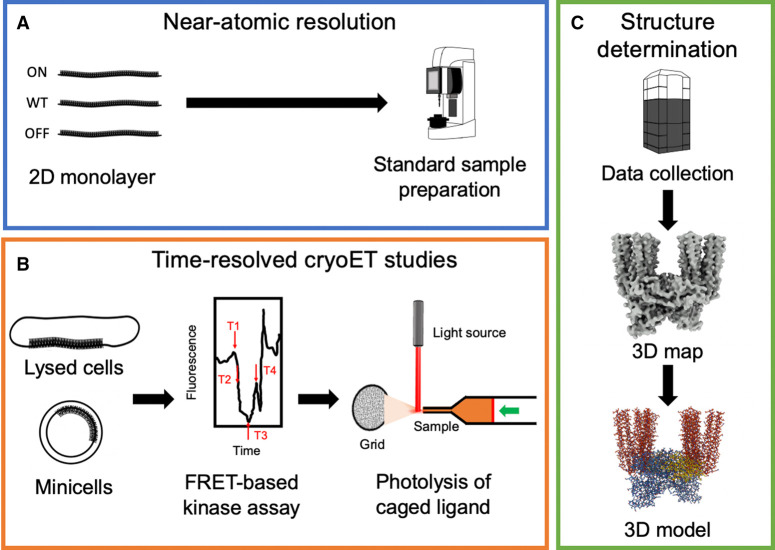
Workflow for near-atomic and time-resolved cryoET studies of chemosensory arrays. (**A**) Near-atomic resolution studies of *in vitro* monolayer systems. After preparing the monolayer systems, which may contain signaling components carrying functional mutations (ON, wild-type, OFF), a standard procedure is used to prepare samples for the microscope. (**B**) A proposed workflow for time-resolved studies of lysed cells and minicells. After preparing the biological samples, a FRET-based kinase assay is used to determine functionally interesting time points based on chemosensory array kinetics (e.g. in response to treatment with a chemoeffector). Grid samples are then exposed to stimulus in a timed fashion (e.g. through the photolysis of a caged ligand) and vitrified at chosen time points to capture signaling states of interest. (**C**) Structure determination for both protocols follows a similar workflow based on a standard cryoET structure analysis pipeline, complemented by computational modeling techniques.

Moving forward, researchers will continue to utilize a wide range of techniques to form a more comprehensive molecular picture of chemotaxis signaling in *E. coli* and other bacteria. CryoET will continue to play a central role in this effort. Forthcoming cryoET investigations will focus on two principal aims: (i) achieving near-atomic resolution structures of the *E. coli* CSU and larger array and (ii) characterizing array structure and dynamics in distinct signaling states. Continued improvements in hardware and image processing combined with the *in vitro* monolayer system, potentially stabilized via strategic mutation, has the potential to provide near-atomistic resolution structures (Figure 4A). Such structures could then be combined with molecular simulation techniques to further provide information on the relative dynamics of each state. Additionally, the more native-like structures emerging from future studies using lysed cells and minicells, although likely to be at a lower resolution, will provide critical conformational information that can more readily be adapted at the biological level. For the most part, the technical and methodological aspects required to carry out the above studies are in place. Another up-and-coming approach for studying complex biomolecular processes is time-resolved cryoEM [83,84]. While still under development for chemotaxis applications, time-resolved cryoET promises to provide a powerful means of achieving unique state-dependent information (Figure 4B). For example, FRET-based kinase assays could be used to identify interesting time points in the array response to a specific chemoeffector. This information could then be used in tandem with the timed chemoeffector-treatment of grid samples (e.g. through the photolysis of caged ligands) to develop a vitrification schedule, down to the millisecond time scale, that captures key signaling states. Ultimately, such time-resolved information can be combined with molecular models to produce movies of chemotaxis signal transduction at near-atomic resolution.

## Perspectives

- Chemotaxis pathways are the best-understood biological signaling systems and serve as a powerful tool for investigating the molecular mechanisms of protein signal transduction. As chemotaxis plays an important role in the infection process of numerous animal and plant pathogens as well as the formation of biofilms, a detailed molecular understanding of chemotaxis signaling would greatly enhance our ability to affect bacterial behavior for medicinal and technological applications.
- We now have a basic understanding of the structure of the *E. coli* chemotaxis machinery, owing in large part to cryoET imaging of chemosensory arrays. At the same time, numerous structural, biochemical, biophysical, and computational approaches have shed light on critical aspects of signaling within the core-signaling unit (CSU) and the extended array. A comprehensive molecular picture of signaling, however, has so far remained elusive, owing to the lack of high-resolution descriptions of array structure and dynamics in distinct signaling states.
- Future cryoET applications will seek to modify existing *in situ* and *in vitro* imaging contexts to provide increasingly detailed images of the CSU in varying signaling states, hopefully achieving near-atomic resolution in certain cases. Molecular modeling and simulation techniques will provide a complementary and independent tool to access high-resolution structural and dynamic detail. Down the road, the development of time-resolved cryoET methodologies should provide millisecond-resolved structural information on native arrays in experimentally controlled signaling states.

## Open Access

Open access for this article was enabled by the participation of University of Oxford in an all-inclusive *Read & Publish* agreement with Portland Press and the Biochemical Society under a transformative agreement with JISC.

## Abbreviations

cryoET: cryo-electron tomography
CSU: core-signaling unit
MD: molecular dynamics simulation
MDFF: molecular dynamics flexible fitting
STA: sub-tomogram averaging
TOD: trimers-of-dimers
